# Perspectives on training in obstetrics and gynaecology during the COVID-19 pandemic: Thematic analysis of trainee responses from a pan-European survey

**DOI:** 10.52054/FVVO.15.3.085

**Published:** 2023-09-24

**Authors:** H Khattak, F Broekhurst, G Topcu, A Horala, M Henriques, H Woodman

**Affiliations:** Tommy’s National Centre for Miscarriage Research, Institute of Metabolism and Systems Research, University of Birmingham, Birmingham, UK; Haaglanden Medical Center, Lijnbaan, The Hague, The Netherlands; Kackar State Hospital, Pazar, Rize, Turkey; Gynecologic Oncology Department, Poznan University of Medical Sciences, Poznan, Poland; Hospital Professor Doutor Fernando Fonseca, Amadora, Portugal; College of Medical and Dental Sciences. University of Birmingham, Birmingham, UK

**Keywords:** Obstetrics and Gynaecology, Postgraduate training, medical education, COVID-19, ENTOG, trainees

## Abstract

**Background:**

Coronavirus disease 19 (COVID-19) has affected many aspects of the lives of medical professionals. Postgraduate training has also been affected and mitigation plans are still ongoing.

**Objective:**

To understand the perspectives of trainees in obstetrics and gynaecology (ObGyn) during the pandemic.

**Materials and Methods:**

A cross-sectional exploratory survey conducted electronically from 20th of April 2020 to 1st July 2020.

**Main Outcome Measures:**

The original questionnaire comprised of 40 questions and a free-text option. The free-stext questions covered five main domains: effect of the pandemic on training, worries about training, acquisition of skills during the pandemic, training period and extensions and responsibilities outside training during the pandemic. The responses to these questions in the survey were analysed using pragmatic thematic analysis.

**Results:**

Trainees felt there was lack of training as well as training opportunities. Some took the pandemic as an opportunity to gain new skills. Trainees were also worried about time in training and uncertainty about extensions. Lastly, many had concerns pertaining to patient care, an inability to contribute to departmental organisation, and dissatisfaction with the implemented policies.

**Conclusion:**

The difficulties in Obstetrics and gynaecology training due to the pandemic need to be mitigated. When planning for reshaping the training programmes to accommodate for the discrepancies caused, trainers need to consider the perspectives of trainees and actively involve them in the decision making, designing and executing future plans.

**What is new?:**

Efforts are currently underway to address the training time lost during the pandemic in Europe. Recognising the paramount importance of providing exceptional care for women and children across the continent, it becomes imperative to consider the valuable perspectives and insights offered by those who represent the future generation of specialists in the field.

## Introduction

The Coronavirus disease 2019 (COVID-19) pandemic led to healthcare systems across the globe having to rapidly re-organise and prioritise emergency care. In order to provide adequate staffing levels for emergency care, many doctors were redeployed from their parent specialty to help the frontline staff. Trainees in obstetrics and gynaecology (ObGyn) were also redeployed and were spending part, if not all, of their working hours, in another specialties (primarily acute medicine and intensive care). The lack of doctors in surgical specialties like ObGyn and prioritisation of emergency care, led to cancellation of outpatient clinics and elective surgical procedures (COVIDSurg Collaborative, 2020; Kasaven et al., 2020; Rimmer et al., 2020; Søreide et al., 2020; Fowler et al., 2021; Spurlin et al., 2021). Understandably, there was a negative impact on postgraduate teaching and training opportunities in surgical specialties worldwide (Balhareth et al., 2020; Bitonti et al., 2020; Adesunkanmi et al., 2021; Aziz et al., 2021; Patil et al., 2021). The pandemic has also had a great impact on the well-being of healthcare professionals (Aljehani et al., 2020; Bhagavathula et al., 2020; Shaw, 2020; Tan et al., 2020; Alshdaifat et al., 2021). ObGyn involves working in a fast-paced and high-pressured environment and the need to reorganise work and accept new responsibilities due to the pandemic has put additional strain on doctors (Usher et al., 2020; World Health Organization, 2020). It has been over three years since the pandemic spread globally and specialties are now looking into finding possible solutions to compensate for the effects on training (Mallick et al., 2021). This may involve redesigning educational programmes to account for lost time and opportunities. Since trainees are affected first-hand, their involvement in such undertakings is essential. To understand how trainees coped with the added pressure of the pandemic and effect on their training, a pan-European electronic survey was conducted. Through the white space free-text questions in this survey, we aimed to explore trainees’ perspectives in relation to the effect of the pandemic on their training.

## Methods

An electronic survey was conducted during the first wave of COVID-19 pandemic in Europe from 20th of April 2020 to 1st July 2020. The survey was written in English and was emailed to representatives of the 35 member countries who were asked to disseminate to trainees in their respective countries. Members of the European Network of Trainees in Obstetrics and Gynaecology (ENTOG) were also invited to take part in the survey using online platforms (social media). Participation in the survey was voluntary. Trainees were asked questions regarding five main domains: effect of the pandemic on training, worries about training, acquisition of skills during the pandemic, training period and extensions and responsibilities outside training during the pandemic. Results of the survey were analysed using pragmatic thematic analysis.

## Data collection

All the data were collected using an electronic questionnaire of 40 questions that were subdivided into four subjects: workload, specialist training aspects in obstetrics and gynaecology, health and safety of the trainee and women’s health and maternal health issues. The results of the quantitative analysis of this survey have already been published (Boekhorst et al., 2021). The survey also consisted of white space free-text option to explore the perspectives of trainees further.

Data was collated in Microsoft Excel. Data analysis was conducted in two stages. One author (H.K.) analysed all the data and the second author (H.W.) verified the extracted themes. Any discrepancies were resolved through discussion until consensus was reached. If discrepancies were not resolved by discussion, a third reviewer (F.B.) was approached.

## Data analysis

Survey responses were analysed using pragmatic thematic analysis. The process of thematic analysis was instructed by Braun and Clarke (2006) and involved the following steps:

1. Data familiarisation- Initial ideas were noted and data were read multiple times to identify ideas.
2. Generating initial codes- The quotes that gave interesting insights were highlighted in a systematic way.
3. Defining and naming interpretive codes
4. Identifying patterns across the data
5. Defining and naming themes

## Results

**Figure 1:**
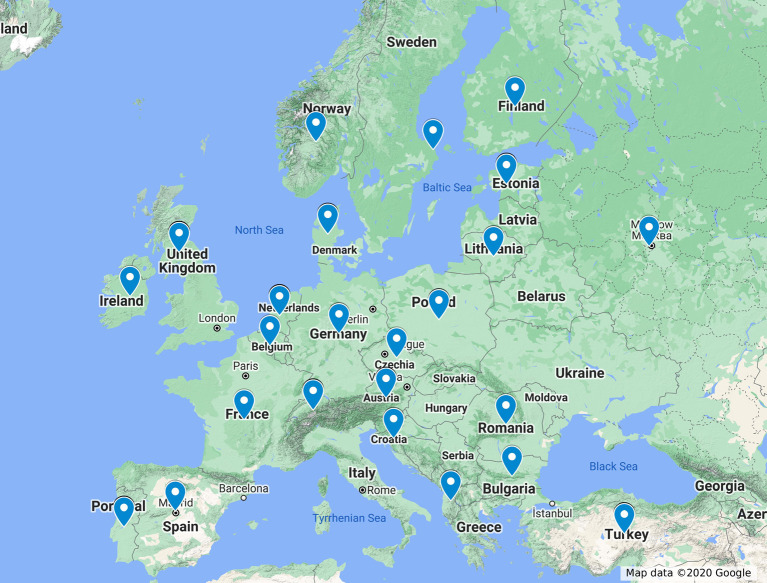
Map representing responses from participating countries.

**Figure 2:**
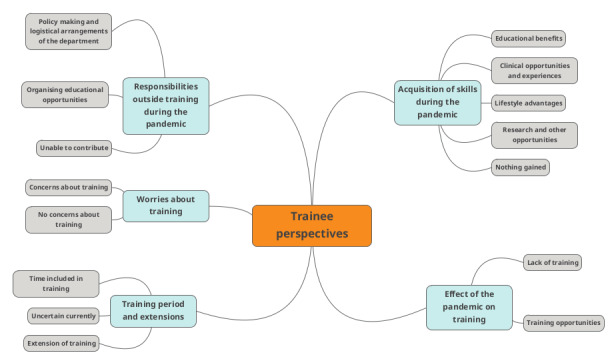
Schematic representation of the generated themes.

Responses from 110 trainees from 25 different European countries were analysed. Figure 1 represents the countries that took part in the survey. Trainee responses and emerging themes are discussed below. Figure 2 provides a schematic representation of the originated themes.

## Effect of the pandemic on training

109 trainees responded to this question. Two main themes emerged: lack of training and effect on training opportunities.

### 3.1.1 Theme: Lack of training

As a whole, trainees felt that there was a greater impact on gynaecological training. Obstetrics care was overall not affected, primarily because most of the obstetrics care is emergency based. Cancellation of elective operations led to a decrease in gynaecological surgeries particularly affecting those trainees with an interest in gynaecology.

These trainees were largely in their later years of training, which also meant that they were worried about finishing their training on time. For those that could finish on time, they felt that they would not be able to practice independently due to lack of exposure to gynaecological surgeries.

‘No effect on obstetric care. Major reduction in all areas of gynaecological care’

‘We have stopped planning surgery in gynaecology, so I have stopped my training in surgery.’

‘[There were] no gynaecological surgeries during the final part of my gynaecological training.’

Being redeployed meant further time away from ObGyn training which further disrupted training opportunities. This again had greater ramifications for trainees in their penultimate and last years of training.

‘Deployment [is] limiting experience.’

### Theme: Training opportunities

As patients were asked to not attend hospital unless absolutely indicated, fewer patients attended. Given scheduled procedures were not taking place either, trainees felt this added further to a severe decrease of training opportunities.

‘Less clinical experience because of a lower number of patients.’

‘Almost all clinical and scheduled procedures are cancelled.’

Some trainees however took it in their stride and found opportunities to develop other skills.

‘For me it has given me the opportunity to attack other parts of my training. For instance, I am growing more in my supervisor role, and I’ve been given the chance to do more things by myself like hysteroscopy (which I was ready for, but the process has been sped up because of fewer doctors needing to be in a room).’

‘I focused more on ultrasound skills during this time, reading and emergency surgeries.’

## Worries about training during the pandemic

Trainees were asked if they had any ‘worries about their training’. All trainees (110) responded to this question with a ‘yes’ or a ‘no’ and 89 expanded their answer further. Sixty-nine trainees answered ‘yes’. Of these 69, 62 provided an answer to the follow up question where they were asked about explaining their response further. Of the 69 trainees who answered yes, 21 were training in the UK, 10 were training in the Netherlands and 4 in Denmark. Broadly, there were two thematic groups: those with concerns about their training and those without.

### Theme: Concerns about training

Trainees expressed concerns about experience and exposure to clinical opportunities.

‘Because it is the end of my studies. And I think I don’t have enough experience to start working on my own.’

‘I am worried about my progress, as I have to change to a university hospital soon and feel I have lost the last months of training in my current hospital regarding O&G competencies.’

‘I am worried I’m getting behind, especially in my surgical skills.’

Trainees also felt that there was a lack of teaching during the pandemic. Understandably some mandatory courses had to be cancelled, especially in the beginning of the pandemic where online delivery of teaching sessions and seminars was not put in place.

‘I am mostly worried about the compulsory training / courses that we have to attend and complete during our 6 years of training. There is a specific order and timeframe at which time we have to complete these. They are all cancelled / postponed.’

### Theme: No concerns about training

Our results showed that 43 of the 110 trainees answered ‘no’ when enquired about worries in relation to training. Of these 43 trainees, 27 provided an answer to the follow up question. The trainees who answered ‘no’ to this question, 6 were training in the UK, 13 in the Netherlands, 4 in Austria.

It was clear from our findings that trainees were pragmatic and already knew that their training would either be postponed, prioritising emergency work, or would be extended. Some trainees chose to pause their training and defer some of their development.

‘We agreed on a pause in gynae study and thus a delay so that I can learn gynae things later on. To be honest, what’s a few months of delay compared to a 6-year study.’

‘My training may be extended but there are other things to worry about.’

Trainees felt that the pandemic was unlikely to have an impact in the longer term and that the extension of training would not necessarily affect their training overall.

‘...since I am only in the first year of training, if the situation starts to go back to normal in the upcoming few months, I think my training will be ok.’

‘Even if I have to train a little bit longer, I don’t think this is a problem.’

## Skills gained or lost as a result of the pandemic

Of the 110 trainees that responded to the survey, 108 trainees provided a response to this question. The qualitative analysis generated five themes; educational benefits, clinical opportunities and experiences, lifestyle advantages, research and other opportunities and no skills gained.

### Theme: Educational benefits

For some trainees the pandemic brought in numerous benefits, especially when it came to attending remote teaching sessions.

‘Increased use of remote learning and meetings [for example] with zoom means I can attend training on non-working days which wasn’t possible before.’

### Theme: Clinical opportunities and experiences

The pandemic resulted in exploring innovative strategies in order to continue to practice medicine safely. Many consultations began to occur virtually using several telecommunication platforms. Trainees felt this was an opportunity to gain further skills such as ‘telehealth’. Furthermore, because of redeployment, sickness and rota changes, trainees on the ground felt more involved in clinical activities, further adding to their experience.

‘triaging of gynae patients, using telehealth.’

‘More chance of performing urgent surgeries due to lack of personnel.’

‘The awareness of the problems that can be first hand handled contactless (i.e., when you can arrange blood analyses without seeing the patient for example regarding infertility or when counselling can be done via phone for example contraception - lots of things to learn about how to make outpatient visit more efficient!).’

### Theme: Lifestyle advantages

The redesigning of rotas and some trainees being redeployed to work on the frontline also brought in flexibility. This helped with having free time that helped with both personal wellbeing of trainees and professional activities such as time for membership examinations.

‘Flexibility.’

‘I have more time to study for the exam and science.’

### Theme: Research and other opportunities

Not all trainees have opportunities to conduct research-related activities as well as to develop other skills. It was felt that the pandemic helped with having such prospects.

‘More involved in the development of guidelines.’

‘More time for research.’

‘I have the chance to improve other competencies, mainly on an organisational level. Example: I have a position in the hospital medical-OMT: a multidisciplinary team managing the outbreak in my hospital.

### Theme: Nothing gained

Not all trainees were optimistic about having time during the pandemic for gaining extra skills. Some trainees felt that being redeployed deskilled them and resulted in loss of training time.

‘Very little [gained]. I have had more than enough time working on a general medical ward prior to working in O&G and this experience has not improved those skills.’

‘Mostly, I lose my training time. I don’t see the gains in a situation where most of the countries are poorly equipped with PPE.’

## Training period and extensions

Of the 110 trainees that responded to the survey, 96 trainees provided a response to this question. The qualitative analysis generated three thematic groups, those who had their training time extended (46 respondents), those who didn’t (11 respondents), and those who are still not certain if it will be extended.

### Theme: Time included in training

Trainees stipulated that at the time of survey there was no inclination towards extension of training. Although they were now covering different aspects of patient care (and not always ObGyn related), some trainees still felt being ‘in training’ but with a different focus.

‘For a large part we are still in training, only the focus is different.’

‘The training will not be extended: it was a decision at national level.’

### Uncertain currently

Some trainees were not sure whether the pandemic will impact their training such that they will require an extension.

‘I don’t know yet, they are busy thinking how to imply.’

‘We haven’t talked about it, but I assume no.’

### Extension of training

A few trainees however showed apprehension and predicted that it is likely their training will be extended.

‘Yes [there has been an effect on training and] it will be extended.’

‘My rotation in another hospital as my obligatory part of residency is postponed.’

‘..delayed CCT (certificate of completion of training).’

## Responsibilities outside training during the pandemic

Of the 110 trainees that responded to the survey, 102 trainees provided a response to this question. The qualitative analysis generated themes relating to trainees contributing to governance and education.

### Theme: Policy making and logistical arrangements of the department

Trainees were involved in roles other than their clinical commitments during the pandemic. This gave some trainees new skills.

‘I wrote all the protocols in the department concerning COVID-19.’

‘I’m scheduling the clinics now.’

‘[I am] involved in deciding what “regular care” can be started with when allowed.’

### Theme: Organising educational opportunities

Some trainees were involved in organising teaching and training sessions in addition to clinical work.

‘Just helping/organizing video call lectures for all the residents, so that we at least keep our knowledge up and running.’

‘As residents we are planning education sessions.’

### Theme: Unable to contribute

A few trainees felt that they were not involved in any of the decision making when it came to reorganising the department, governance or tackling the challenges of the pandemic. They felt they were not allowed to have their opinions heard.

‘Not at all. Decisions are made at the top and communicated to us.’

‘Not allowed to speak up even when its constructive suggestion.’

## Discussion

Through this study we aimed to highlight trainees’ perspectives on various issues that came to surface. Overall trainee morale was low with most trainees having negative sentiments towards teaching and training opportunities. Lack of gynaecological training opportunities meant that for some trainees their training period had to be extended. The findings related to training time and extension in our study are synonymous with responses collected in the quantitative questions of the survey. For instance, over 60% of respondents were worried about meeting training objectives in their curriculum, which was reflected as trainees being uncertain regarding the time it would take to complete the remainder of their training and whether they would actually have the necessary skills required to practice independently upon becoming a specialist (Boekhorst et al., 2021).

Some trainees also felt that they were not involved in the decision making of how clinical care and training were organised during the pandemic. It was noted that decisions were mostly made at a senior level and trainees were expected to oblige. For some it also meant they felt unable to make a meaningful contribution to their departments. This was felt to be detrimental to both their self-esteem and their professional development. Doctors are increasingly becoming burnt out across most specialties (Shanafelt et al., 2009; Pulcrano et al., 2016; West et al., 2016; Levin et al., 2017; RCOG, 2018). Our study findings further demonstrate the increasing prevalence of physician burnout with ObGyn trainees.

Additionally, the pandemic has brought with itself opportunities that were not previously considered. From the quantitative responses, 72% of trainees who had previously lacked experience in management reported to have gained extracurricular skills such as crisis management, rota coordination and guideline development. (Boekhorst et al., 2021) Furthermore, flexible working, which has not historically been applied to clinical work, was finally adopted during the pandemic. For instance, the ‘work from home’ option was previously not thought applicable to clinicians. Where relevant and feasible, the option of being able to conduct clinics and administrative tasks from home may prove beneficial for trainee wellbeing.

Furthermore, due to lack of training opportunities, great emphasis is now placed on strategies to recover training, which includes active participation in simulation training. (Zimmerman et al., 2021) Our study has clearly highlighted the lack of surgical training opportunities for ObGyn trainees and how they pandemic has made the situation worse.

Although simulation training in ObGyn training has been implemented for decades, there is still the need to incorporate high-fidelity simulators, including virtual reality, to improve surgical skills of trainees. Trainers are therefore urged to consider the use of hi-fidelity simulation including virtual reality when making training recovery plans. Additionally, trainees could also potentially benefit from encouragement and funding towards completing formal accreditation courses that aids surgical training, such as the Gynaecological Endoscopic Surgical Education and Assessment (GESEA) programme. (GESEA, 2022)

Currently there is a lack of international consensus on how to tackle ObGyn training affected by the pandemic. Although training differs across Europe, our study shows that the main concerns were broadly similar across nations. Both the European network of trainees in O&G, European Board and College of O&G and specialist societies that are involved in improving gynaecological training, need to form a consortium to address training needs.

Trainees are our future specialists. The workforce climate is evolving rapidly, and it is therefore imperative that our trainees’ perspectives are taken into consideration when deciding on strategies to recover from the pandemic. This will particularly benefit trainees in their final years of training who will soon become independent specialists. Despite the negative impacts of the pandemic on training, some trainees felt that rota rearrangement and the focus on emergency services proved to beneficial, particularly with regard to flexible training hours. This was complemented with trainees being exposed to managerial and leadership opportunities as well as gaining research skills. The use of telemedicine and innovative remote working opportunities was found to be very useful by trainees and many thought this should be implemented permanently. Furthermore, teaching was mostly imparted using digital platforms, which increased attendance and access to teaching.

### Limitations

One limitation of the survey was that not all European countries were represented. There perhaps was also a selection bias given the survey was in English and only English-speaking trainees took part. This may have further put some trainees off from responding to the open-ended questions.

### Strengths

To the best of our knowledge, our study is the first study that explores the perspectives of how European ObGyn trainees felt during the pandemic. Despite differences in training, it seemed that the views and perspectives of trainees resonated across all European nations. Many other specialties have explored the impact of the pandemic on training however none of the studies have focused on trainees’ perspectives through a a qualitative perspective.

## Recommendations

Based on our study, we suggest the following recommendations:

- Consider trainee involvement in leadership and management decisions (from all year groups and not just those in their last years of training) to boost their confidence and readiness for independent specialist roles.
- Incorporate flexible working for trainees where possible to promote their well-being, work-life balance, learning opportunities, and ultimately improving their contribution to better patient care.

### Areas for future investigation:

Further investigation and research are required to improve ObGyn training post the pandemic, specifically in establishing an international consortium focused on addressing the challenges trainees encounter and finding ways to improve training opportunities that were missed. Collaboration among experts in Postgraduate Medical Education is essential on a global scale to address these concerns comprehensively. Additionally, incorporating telemedicine into the curriculum is crucial, considering its increased utilisation since the pandemic. This mode of conducting consultations should be explored, particularly for trainees with longstanding health conditions who may benefit from working remotely. Furthermore, the adoption of digital technologies, such as artificial intelligence and virtual reality, in teaching and training, deserves attention.

## Conclusion

The COVID-19 pandemic has greatly affected training across specialties and has understandably caused concerns amongst the ObGyn doctors in Europe. The difficulties in OBGyn training caused as a result of the pandemic need to be mitigated. When planning for reshaping the training programmes to accommodate for the discrepancies caused, trainers need to consider the perspectives of trainees and actively involve them in the decision- making, designing and executing of reforms.
